# Immune Reactions in Major Types of Oncological Treatment

**DOI:** 10.3390/ijms241411257

**Published:** 2023-07-09

**Authors:** Patrycja Kozubek, Julia Wołoszczak, Krzysztof Gomułka

**Affiliations:** 1 Student Scientific Group of Adult Allergology, 50-369 Wrocław, Poland; julia.woloszczak@student.umw.edu.pl; 2 Clinical Department of Internal Medicine, Pneumology and Allergology, Wroclaw Medical University, 50-369 Wrocław, Poland; krzysztof.gomulka@umed.wroc.pl

**Keywords:** adverse reactions, side effects, biological agents, chemotherapy, desensitization

## Abstract

In recent years, there has been a noticeable development in oncological treatment, including chemotherapy and biological treatment. Despite their significant effectiveness, they are not free from side effects, such as allergic and dermatological reactions. These reactions can vary in severity and outcome, including potential death. Examples, among others, are type I-IV hypersensitivity reactions of various origins and skin reactions including rashes, itching and redness, but also severe cutaneous syndromes. Due to the therapy used, these may include Stevens–Johnson syndrome, toxic epidermal necrolysis, drug rash with eosinophilia and systemic symptoms, drug-induced hypersensitivity syndrome and acute generalized exanthematous pustulosis. In some cases, it is necessary to interrupt therapy, which may result in a poorer outcome and shorten the patient’s survival. This paper reviews various types of research documents published since 2016. It aims to systematize the latest knowledge and highlight the need for further research into ways to avoid adverse reactions.

## 1. Introduction

Chemotherapy and biological treatment are examples of basic therapies used in oncological treatment. Their use brings good results in many types of cancer; however, it is connected to side effects [1,2]. An important example is immune-related adverse effects (irAEs), which range from mild rashes to severe, life-threatening anaphylaxis [3]. The type of reaction induced is closely related to the drug or drug combination used [4]. A noticeable side effect is dermatological changes, from mild, such as itching, rash and eczema, to potentially lethal forms. Examples are severe cutaneous adverse reactions (SCARs), including Stevens–Johnson syndrome (SJS) and toxic epidermal necrolysis (TEN) [5,6]. In some cases, skin reactions are associated with a better response to treatment [6,7]. Cancer therapy also has a significant impact on the quality of life of patients. They struggle with a sense of discomfort associated with pain and itching [8]. 

The main aims of this work were to emphasize the variety of immunological side effects in oncological treatment and to outline the need for future research. In the future, researchers should focus on ways to prevent side effects and treat the symptoms that have already arisen with methods that would not involve discontinuation of therapy.

This article is an attempt to review and systematize the latest knowledge. For its writing, a literature review of the PubMed, ResearchGate and Google Scholar databases was conducted with the keywords hypersensitivity reactions, adverse reactions, chemotherapy, biological agents and drug rash and the date of publication from 2016 to 2022. Only English-language literature was used. The obtained information was analyzed and systematized.

## 2. Biological Drugs

Biological drugs are substances synthesized by living organisms or made from a substance produced by a living organism. In recent years, a significant increase in the frequency of using this group of drugs in the treatment of many diseases, including oncology, has been observed. They are used in the treatment of, among others, breast, lung, bladder, cervical, head and neck, gastrointestinal and kidney cancers, as well as leukemias and lymphomas. Biologics include vaccines, hormones, growth factors, immunoglobulins, monoclonal antibodies (mAbs) and others [3,9].

### 2.1. Classification

Biological drugs in cancer therapy can be divided into the following groups: human, humanized, murine and chimeric. Different groups have a distinct ability to cause adverse reactions, including immune-related adverse effects (irAEs) [10,11]. Side effects are proportionately less common than with the use of traditional chemotherapy and systemic side effects are less noticeable. Attention is paid to immune reactions occurring at the beginning or during the use of biological drugs [12]. Some skin reactions have been shown to be associated with better treatment outcomes and better survival [6]. Examples include the occurrence of skin rash as a predictive marker of response to cetuximab treatment in metastatic colorectal cancer [7]. A similar correlation occurs with the use of epidermal growth factor receptor (EGFR) inhibitors in patients with non-small cell lung cancer and nivolumab or pembrolizumab in patients with melanoma [6].

The side effects of mABs are classified, according to Pichler and the mechanism of action, into:

- type alpha—high cytokine and cytokine release syndrome (CRS), infusion-related reaction (IRR);
- type beta—hypersensitivity, which can be divided into: IgE-mediated, IgG or T lymphocyte;
- type gamma—immune imbalance syndrome;
- type delta—cross-reactivity;
- type epsilon—non-immunological side effects [1,13].

Typical hypersensitivity reactions to mAbs include the following phenotypes: IRR and CRS, type I (IgE/non-IgE), type III and type IV (Gell and Coombs classification) and all beta reactions [1]. IRR and CRS on mAbs may occur during the first infusion and usually present as symptoms of varying severity. Hot flushes, chills, fever, tachycardia, hypertension, dyspnea, nausea, vomiting and syncope may occur. Type I reactions to mAbs, such as those seen with rituximab and cetuximab, may manifest as flushing, pruritus, urticaria, dyspnea, hypotension and anaphylaxis. HSR type III has been described for infliximab, etanercept and adalimumab. Most often, the classic triad of fever, joint pain and rash appears after application. Type IV delayed hypersensitivity reactions to mAbs including rituximab may range from mild maculopapular rash to severe reactions (Stevens–Johnson syndrome, toxic epidermal necrolysis). They usually appear 12 h to several weeks after exposure. IgE-mediated reactions do not occur on the first exposure, except for cetuximab, which, due to cross-reactivity with galactose-α-1,3-galactose (alpha-gal), may cause hypersensitivity on the first administration [4,14].

The severity of HSRs can be assessed according to the modified Brown classification (listed in Table 1). Grade I is the most common (63% of cases).

### 2.2. Clinical Picture

Molecularly targeted therapies and immunotherapies are associated with a wide range of dermatological adverse events resulting from common signaling pathways involved in malignant growth and normal homeostatic functions of the epidermis and dermis. Dermatological toxicity is often associated with the use of biological drugs. It involves damage to the skin, oral mucosa, hair and nails. Acne-like rash is the most common side effect [5].

Checkpoint inhibitors (CPIs) are used to treat cancers of the kidney, lung, bladder and liver, among others. The incidence of side effects caused by checkpoint inhibitors ranges between 54% and 76% and is higher with the combination of ipilimumab plus nivolumab [15,16]. iRAe most commonly presents as a nonspecific maculopapular rash, but also pruritus, eczematous lesions, lichenoid dermatoses and vitiligo. Less common side effects include psoriasis-like dermatoses, bullous disorders and severe cutaneous adverse reactions (SCARs) [5,17].

Another group of biological drugs that cause skin reactions are immune checkpoint inhibitors (ICIs), which include, among others, CTLA-4, PD-1 and PD-L1 inhibitors. Maculopapular eruptions occur in up to 60% of patients treated with CTLA-4 inhibitors, histopathologically showing perivascular dermatitis. Perivascular dermatitis may also occur during PD-1 blockade, sometimes with eosinophilia. Patients show varying degrees of pruritus, erythematous macules and dome-shaped papules, some of which coalesce into macules and plaques [18].

VEGF inhibitors, for example Lenvatinib, may lead to stomatitis, palmar–plantar erythrodysesthesia syndrome (PPES) and other side effects associated with biological drugs [19].

For BRAF inhibitors (for example, vemurafenib and dabrafenib), the main cutaneous side effects are neoplastic growths, mainly cutaneous squamous cell carcinoma, keratoacanthoma and papillary keratosis. Inflammatory reactions also occur, for example, rashes, photosensitivity reactions, palmar–plantar skin reactions and changes in pre-existing pigmented lesions [5,9].

The EGFR inhibitors (EGFRi) erlotinib, gefitinib, cetuximab, necitumumab or panitumumab are commonly used to treat advanced lung, colorectal, breast and head and neck cancer. The MEKs trametinib, cobimetinib, binimetinib and selumetinib are used to treat several cancers, including melanoma, colorectal cancer, non-small cell lung cancer, pancreatic cancer and hepatocellular carcinoma. Despite their clinical utility, EGFRi and MEKi are associated with significant skin toxicity, the most common being papulopustular eruptions [20,21]. Other cutaneous adverse events for EGFRi include dry skin, hair and nail changes, paronychia and mucositis, and for MEKi include morbilliform eruption, dry skin, paronychia, alopecia and hyperpigmentation [20].

**Figure 1:**
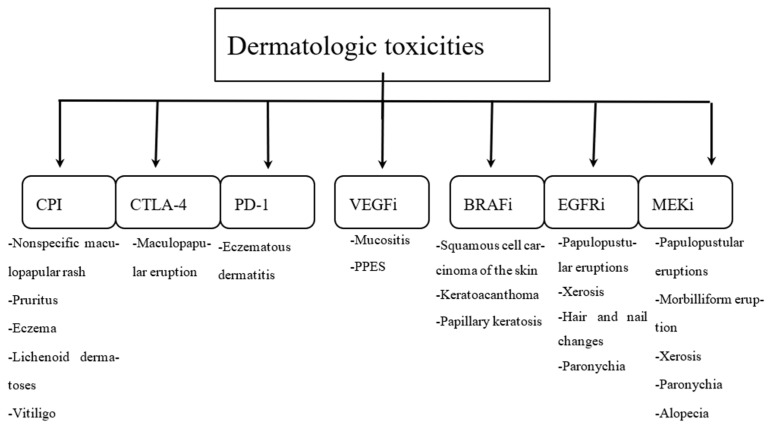
Selected examples of dermatologic toxicities [5,9,15,16,17,18,19,20,21].

Selected examples of dermatologic toxicities are shown in Figure 1.

### 2.3. Severe Immunological Reaction

Severe immune reactions caused by biological drugs include severe cutaneous adverse reactions (SCARs): Stevens–Johnson syndrome (SJS)/toxic epidermal necrolysis (TEN), drug rash with eosinophilia and systemic symptoms (DRESS)/drug-induced hypersensitivity syndrome (DIHS) and acute generalized exanthematous pustulosis (AGEP). They can potentially be fatal [6]. The clinical picture of DRESS or DIHS is usually complex and includes fever, skin lesions with typical skin eruptions (e.g., eosinophilia), lymphadenopathy and internal organ involvement (e.g., liver, kidneys and lungs). The RegiSCAR criteria used to diagnose potential cases of DRESS require at least three of the following: fever above 38 °C, enlarged lymph nodes in at least two areas, involvement of at least one internal organ or blood abnormalities. Biologics that can cause it include imatinib, vemurafenib, bortezomib, ipilimumab and vemurafenib [6,22]. AGEP, on the other hand, is characterized by the sudden onset of mainly small non-vesicular erythematous pustules associated with systemic changes such as fever and neutrophilia. AGEP can be caused by, for example, imatinib, sorafenib, gefitinib, vemurafenib and ipilimumab. Cases of SJS/TEN have been reported with imatinib, rituximab, vemurafenib, nivolumab, pembrolizumab, ipilimumab and some EGFR inhibitors [6].

### 2.4. Summary

As biological agents’ use frequency increases, a variety of adverse reactions can occur with immune-related adverse effects among them. These reactions can arise at the beginning of or during therapy. Although the list of symptoms is long and represents all types of Gell and Coombs classifications for specific drugs, most of the cases are recognized as mild ones. The following belong to the group of most common culprit drugs: checkpoint inhibitors, immune checkpoint inhibitors, VEGF inhibitors, BRAF inhibitors and EGFR inhibitors. Among the immune-related adverse effects caused by biological drugs, there are also severe immunological reactions including AGEP, SJS/TEN and DRESS, indicated by a long list of group representatives.

## 3. Classical Chemotherapeutics

Chemotherapeutics are one of the basic components of oncological regimens, both in the form of adjuvant, neoadjuvant or palliative therapies and strategies combined with drugs from other groups. The possibility of using cytostatics in oncology with the entire spectrum of applications is limited by a number of side effects. Most of these are due to significant toxicity, but the administration of some drugs is also associated with an increased risk of hypersensitivity.

### 3.1. Classification

The main groups of chemotherapeutics include:

- alkylating agents (platinum derivatives—cisplatin, carboplatin, oxaliplatin; cyclophosphamide, ifosfamide, chlorambucil, procarbazine, chlormethine);
- antimetabolites (methotrexate, pemetrexed, cytarabine, gemcitabine, fluorouracil);
- mitosis inhibitors (taxanes—paclitaxel, docetaxel; vinca alkaloids—vincristine, vinblastine);
- cytostatic antibiotics (anthracyclines—doxorubicin, daunorubicin, idarubicin, epirubicin; bleomycin, mitomycin, mitoxantrone and amsacrine [23].

The drugs most commonly implicated as causes of HSRs include carboplatin, cisplatin, paclitaxel and docetaxel [2,24]. Mainly type I HSRs, IgE-mediated or not IgE-mediated, are described, while other mechanisms of hypersensitivity, mainly types III and IV, may be involved. Symptoms are variable and difficult to predict, especially in the case of other non-immunological adverse reactions, polytherapy and comorbidities [23,25,26].

### 3.2. Clinical Picture

Carboplatin and cisplatin are mainly used in treatment regimens for ovarian, lung and head and neck cancers, while oxaliplatin is mainly used as first-line therapy for colorectal cancer. It is estimated that hypersensitivity reactions occur in 8–16% of the population of gynecological patients receiving anticancer treatment [26,27]. Type I IgE-mediated HSR predominates, with the first symptoms of either agent occurring most frequently in the eighth or ninth cycle of treatment (usually corresponding to 2.3 cycles after relapse) [27]. As the number of cycles increases, the risk of HSRs increases—approx. 27% risk is found after the seventh cycle [25]. Other risk factors for hypersensitivity after the administration of platinum groupings have been described, such as the maximum dose of carboplatin ≥650 mg, the length of the interval between successive doses, history of allergic reactions to drugs, premenopausal period and unexplained exposure of atopic-prone patients [26]. Reactions are immediate and occur up to several hours after dosing, although, particularly with carboplatin, a late reaction is possible, including drug fever occurring up to three days after dosing [25,27,28]. The predominant symptoms are rash, urticaria, pruritus, skin burning and edema, together with abdominal cramps and diarrhea, which may progress to life-threatening anaphylaxis (bronchospasm, tachycardia, hypotension, convulsions) [29]. Carboplatinum is the most common cause of anaphylaxis compared to other platinum derivatives [24]. Cases of immune-related hemolytic anemia and thrombocytopenia complicated by bleeding have been reported with oxaliplatin. Severe skin hypersensitivity reactions such as SJS/TEN have not been reported. In the literature, there are cases of cross-reactions between platinum salts, confirmed by positive skin tests, which make it impossible to substitute the drug causing hypersensitivity. Their mechanism is not fully understood. It is theorized that cross-reactions with carboplatin and oxaliplatin are due to the presence of a common epitope (nitrogen-platinum-oxygen-carbon-oxygen-carbon (N-Pt-O-CO-C) chain) in both compounds responsible for causing the reaction. Due to the lack of a suspected epitope in the cisplatin compound, it does not cross-react; therefore, according to previous studies, it can be safely used in patients with HSRs after the use of oxaliplatin and carboplatin [29,30].

Taxanes are used in gynecological oncology and the treatment of lung and breast cancer. HSR type I can be both IgE-mediated and non-IgE-mediated—the reaction occurs after the first or second administration of the drug. Immediate hypersensitivity reactions to paclitaxel and docetaxel occur in approximately 10% of patients despite premedication and are severe in 1%. Symptoms of erythema, shortness of breath or hypotension occur up to 5 min after administration. Severe paclitaxel-induced anaphylaxis is not uncommon, although some researchers consider a higher incidence of carboplatin-induced anaphylaxis. After the resolution of mild symptoms, treatment with paclitaxel can usually be continued. Re-administration of the drug after anaphylaxis is considered to be too dangerous [24]. SJS/TEN may also be severe with acute interstitial pneumonia and subacute cutaneous lupus erythematosus [27].

Chemotherapy can also lead to non-immune skin reactions. Cutaneous adverse drug reactions (CADRs) or cutaneous adverse events (CAEs) have a diverse morphology that often mimics other disease entities [31]. Classic chemotherapeutic agents in CAEs in systemic oncological therapies studies were the group of drugs most often causing skin lesions (46.2%). In particular, this applies to paclitaxel, capecitabine, carboplatin and bleomycin. The main ones are subungual and periungual changes, hyperkeratosis, papulopustular rashes, acne-like rashes and erythema [32]. CAEs can present as multiple syndromes. Capillary leak syndrome (CLS) is caused, for example, by doxorubicin and gemcitabine, an acute state of generalized edema associated with hypoproteinemia, rapid rise in hematocrit and critical hypotonia. Possibly associated with a temporary increase in plasma concentrations of inflammatory mediators, it is potentially life-threatening. The already mentioned hand–foot syndrome (HFS) can be induced both by the action of biological drugs and chemotherapeutic agents: cisplatin, cytarabine, capecitabine, doxorubicin, 5-fluorouracil and etoposide, paclitaxel and docetaxel [23,31,32]. The latter one is also suspected of inducing acute generalized exanthematous pustulosis (AGEP) and its more common form, localized exanthematous pustulosis (ALEP) [31]. Neutrophilic eccrine hidradenitis (NEH) is an erythematous, edema–papular lesion that is localized or diffuse, most often involving the trunk. The lesions usually appear a week or two after the start of treatment, accompanied by fever and neutropenia, in response to administration of mainly cytarabine and daunorubicin [23].

### 3.3. Severe Immunological Reactions

Severe immune reactions in chemotherapeutic therapy are rare, but cases of DRESS have been reported—with the use of chlorambucil or SJS/TEN in therapy with alkylating drugs (bendamustine, chlorambucil, cyclophosphamide, mechlorethamine), antimetabolites (capecitabine, cladribine, cytarabine, fludarabine, gemcitabine, pemetrexed) and cytostatic antibiotics (bleomycin, dactinomycin, doxorubicin). SJS/TEN is most often manifested on the 4th to 28th day after administration of the drug with the occurrence of bullous, atypical and confluent lesions, detachment of the epidermis over a large area of the body and inflammation of the mucous membrane. Prolonged fever, respiratory distress and acute renal failure may also be present. Mortality rates in the general population range from 1 to 5% for SJS and 25 to 35% for TEN. In severe HSRs, re-administration of the triggering drug is strongly contraindicated, especially when substitution is possible [23]. Severe immune reactions may also include Sweet’s syndrome, also known as acute febrile neutrophilic dermatosis. Clinically, it is manifested by the sudden appearance of painful erythematous plaques or nodules accompanied by acute fever and leukocytosis. Pustular, bullous and necrotic variants have also been described. The central nervous system, internal organs and the musculoskeletal system may be involved. Chemotherapeutics causing Sweet’s syndrome are drugs from the group of antimetabolites—azacytidine, capecitabine, decitabine and gemcitabine [23,33]. However, it is more often idiopathic or paraneoplastic, particularly in acute myeloblastic leukemia [34].

### 3.4. Summary

Chemotherapeutics are basic and widely used elements of antineoplastic therapy, charged with significant toxicity and hypersensitivity risk. Usually, observed reactions are related to the effects of platinum salts, taxanes and cytostatic antibiotics therapy. The most common is the type I reaction of Gell and Coombs’ classifications. There are also a large number of non-immune skin reactions related to chemotherapeutics that can occur during therapy, like capillary leak syndrome or hand–foot syndrome. Severe immunological reactions occur especially during the use of certain alkylating agents, antimetabolites and cytostatic antibiotics.

## 4. Therapeutic Options

### 4.1. Desensitization

Desensitization is a procedure that allows patients to safely administer a drug for which an HSR has previously occurred. It is of particular importance as it enables the continuation of the therapy of first choice in oncological treatment, when the change of drug is associated with a high risk of therapy failure, side effects and, consequently, a negative impact on the patient’s survival and quality of life [35,36]. The main goal of desensitization in chemotherapy is to maintain temporary tolerance to the drug, which is possible by gradually administering drug doses at specified intervals [37,38,39]. In vitro models have shown that by using subthreshold drug doses and successively increasing doses at intervals, it reduces the allergen reactivity of sensitized mast cells. Dividing the optimal dose into 11–16 incremental doses, starting from 1/1000 of the target dose, and delivering them at appropriate intervals to mast cells inhibits the secretion of β-hexosaminidase by mast cells and, consequently, inhibits the release and production of immune response mediators [35,38]. Thus, it enables the use of a desensitization procedure for mast cell-mediated hypersensitivity reactions that are both IgE-mediated (mainly in the case of platinum derivatives) and non-IgE-mediated (other classes of chemotherapeutics and biologics) [38,40,41]. Indications and contraindications for desensitization are listed in Table 2.

#### 4.1.1. Patient Qualification and Risk Assessment

The decision to undertake desensitization—in the case of oncological drugs, in the rapid drug desensitization (RDD) procedure—is based on the clinical picture, medical history and risk assessment, in cooperation with oncologists and allergists. The main tool for assessing the occurrence of HSRs is the drug provocation test (DPT), involving the controlled administration of the culprit drug. This is now the gold standard for confirming or ruling out hypersensitivity, especially when skin and serum IgE results are negative, unavailable or not validated, and in situations where the patient is taking more than one drug [38,42,43]. Patients with a negative test result are eligible for continued standard administration of the drug suspected of causing the reaction [36]. The use of skin testing (ST) remains unclear due to the potentially low usefulness, especially in the case of non-IgE-inducing drugs, hence the low use of skin testing in the case of taxanes [44]. Usefulness has been proven with HRS induced by platinum salts, which most often cause an IgE-mediated reaction, such as carboplatin and oxaliplatin. However, the sensitivity of the tests also varied significantly between studies (26–100% for oxaliplatin, 66–100% for carboplatin) [40,45]. Nevertheless, they can be a helpful tool in identifying patients at low risk of developing HSRs and, on this basis, selecting the appropriate desensitization protocol [40]. Skin testing is not performed on patients who have received antihistamines in the last 7 days or who have developed dermographism. To reduce false negatives, skin tests are performed two weeks after the initial hypersensitivity reaction [46]. Determination of biomarkers such as tryptase, cytokines (mainly IL-6) and IgE or the basophil activation test (BAT) may be helpful in the assessment of HRS phenotypes and endotypes, but most of them require further research and standardization and are not used in clinical practice [39,47,48,49,50,51].

#### 4.1.2. Procedure

The desensitization procedure requires continuous exposure to the drug; therefore, it involves the implementation of multi-stage protocols, assuming the administration of drug solutions of different concentrations with increasing doses and flow rates [35,52]. It is necessary to maintain constant intervals of 15–20 min in order to stabilize the pharmacokinetics of the drug. The most common is the 12-step protocol with three bags of solution. Most protocols allow the full therapeutic dose to be administered in 4–12 h, although this may be extended in the event of a breakthrough reaction (BTR) [44,45,52,53]. The procedure requires individualization—in patients at high risk of anaphylaxis, 16- and 20-step protocols are proposed to minimize it, while in patients with HSR grades I and II, a 12-step protocol is most often implemented [51,54]. Several years of research on the single-bag protocol show that it is possible to speed up and simplify the procedure while maintaining the effectiveness and safety of the three-bag protocol, which could reduce the risk of errors, reduce costs and increase the availability of desensitization [42,44].

Most publications describe routine premedication with H1 and H2 receptor blockers, supported by other drugs depending on the symptoms, such as benzodiazepines, NSAIDs and opioids, as well as aspirin and montelukast. It has been proven that premedication with H1 and H2 blockers can reduce the severity of BTR more than methylprednisolone [38,55,56,57]. Patients with severe reactions, highly positive STs and a history of chronic urticaria are also treated with oral antihistamines [44]. Currently, most publications recognize the use of glucocorticoids mainly as an antiemetic or as part of cancer therapy. The use in premedication is disadvantageous because these drugs are less effective than aspirin or montelukast while carrying serious side effects, as well as masking the onset of BTR during desensitization [38]. Some sources report that steroids do not have a negative effect on overall survival in patients with hypersensitivity to platinum salts [58]. In a 2022 study, the use of steroids in patients during RDD (but not as a part of premedication) was statistically significant in reducing the incidence of moderate to severe BTR, and this preventive effect was more noticeable with Platinum [59].

Most RDDs are without BTR. In cases with BTR, reactions are usually mild (58–73%), but moderate to severe BTRs are most frequently reported [41,47,60]. Reactions occur in the last stages of RDD, and initial reactions at low drug doses indicate a very high patient reactivity. The desensitization procedure in the event of severe BTR must be stopped or postponed in order to review the protocol, indications and premedication used [38].

The results clearly show the high success rate and low complication rate of the procedure. In combination with maintaining the effectiveness of the drug causing hypersensitivity, RDD is an excellent choice in cancer therapy after HSRs. However, the effectiveness and safety of the method is directly related to the presence of trained staff, good cooperation of the team of oncologists and allergists who are experts in desensitization and an individual approach in creating the RDD protocol for each patient [38,53].

### 4.2. Symptomatic Treatment

Patients who experienced hypersensitivity reactions during therapy should be provided with symptomatic treatment for particular manifestations.

Cutaneous manifestations of HSRs like papulopustular, acneiform or morbilliform eruptions and hand–foot syndrome can be managed with topical antibiotics, corticosteroids and emollients.

For oral mucositis, treatment includes proper oral hygiene and avoiding mint-flavored toothpaste or alcohol-containing mouthwash to avoid irritation of the mucus. In painful ulcerations, topical corticosteroids or topical lidocaine are recommended. Side effects of topical steroids are rare. They are mostly a burning and stinging sensation, worsening of inflammation, thinning of skin, dermatitis or acne. Similarly, the side effects of topical lidocaine use include blistering, irritation and itching and dry, scaly skin. Tachycardia and fever may also occur in rare cases [19].

Severe immunological reactions like DRESS require systemic corticoids, with topical corticoids and oral antihistamines in addition in cutaneous manifestation.

SJS/TEN most importantly requires discontinuation of treatment, which can affect the therapy process and exposes patients to the risk of side effects and low efficiency of the replacement drug. Supporting life functions, suitable nutrition and pain management are also required during severe HSRs facing the risk of multi-organ failure [23].

Carboplatin-related drug fever, a delayed HSR, can be treated if necessary with nonsteroidal anti-inflammatory drugs. Cardiotoxicity and gastric toxicity risk should be considered, especially during therapy with agents of similar properties [28].

In pruritus, management options include gentle skin care, topical antipruritic agents and steroids. In cases not controlled by topical treatment (grade 2 and 3), oral antihistamines, GABA agonists and antidepressants may be necessary. Typical side effects to these systemic medications are sedation, psychomotor impairment and blurred vision after antihistamine and GABA agonist administration, and anxiety, fatigue, dizziness, insomnia, blurred vision and constipation due to antidepressant use [15].

### 4.3. Summary

Desensitization enables patients to continue therapy after hypersensitivity reactions, avoiding further difficulties of immunological reactions, side effects and risk of therapy failure due to changes of medications. The main tool of assessment is the drug provocation test, which confirms or rules out the possibility of hypersensitivity in patients. Other methods like skin testing, BAT and levels of biomarkers are not in common use. The procedure involves multi-stage protocols corresponding to patients’ state and allergy history. It requires applicable premedication and is highly dependent on qualified medical staff. Desensitization presents a high success rate and low risk of complications, which makes it an effective and safe solution to the problem of hypersensitivity in therapy.

Early symptomatic treatment in HSRs depends on the grade of symptoms and usually consists of topical agents for cutaneous manifestation. or symptoms above grade 2, mainly corticosteroid systemic therapy and also pain, fever and pruritus management components are involved. In severe cases, discontinuation of oncological treatment is inevitable.

## 5. Discussion

The introduction of molecularly targeted therapies and the replacement of classic chemotherapeutics with substances of reduced toxicity make it possible to improve the quality of therapeutic processes and their effectiveness and safety. However, patients still experience side effects related to hypersensitivity and high cytotoxicity. They show a wide range of symptoms varying in severity and impact on the quality of life. Thanks to the implementation of premedication, desensitization and symptomatic treatment of side effects, it is possible to continue oncological therapy in most cases. In severe cases of systemic or skin reactions, it may be necessary to suspend drug usage, reduce doses or replace the drug. These actions are associated with a possible negative impact on treatment results and survival. It is necessary to further expand the knowledge on adverse immune reactions and ultimately create guidelines that allow for uninterrupted therapy.

## Figures and Tables

**Table 1:** Modified Brown classification for hypersensitivity reaction severity [4].

Grade	HSR severity
I (mild)	Reactions limited to 1 organ system, typically skin
II (moderate)	Reactions involve ≥ 2 organ system without change in vital signs
III (severe)	Reactions involve ≥ 1 organ system with vital signs changes such as oxygen desaturation, hypotension, seizure, throat closure, loss of consciousness

**Table 2:** Indications and contraindications for desensitization [35].

Indications	Contraindications
Type I hypersensitivity reactions (IgE-mediated, mast cell-mediated)	Severe skin drug reactions (SJS/TEN, DIHS/DRESS, AGEP)
Type IV hypersensitivity reactions (excluding severe skin reactions)	Type II hypersensitivity reactions (cytotoxic)
There is no alternative treatment with similar effectiveness and without side effects	Type III hypersensitivity reactions (sickness-like serum)

SJS—Stevens–Johnson syndrome, TEN—toxic epidermal necrosis, DIHS—drug-induced hypersensitivity syndrome, DRESS—drug reaction with eosinophilia and systemic symptoms, AGEP—acute generalized exanthematous pustulosis.

## Data Availability

Data sharing is not applicable as no datasets were generated or analyzed in the current study.
